# Dihydrokoumine, a dual-target analgesic with reduced side effects isolated from a traditional Chinese medicine

**DOI:** 10.1016/j.jare.2024.10.011

**Published:** 2024-10-24

**Authors:** Dian Liu, Jixia Wang, Tao Hou, Yan Zhang, Han Zhou, Yaopeng Zhao, Liangliang Zhou, Cuiyan Cao, Yanfang Liu, Xinmiao Liang

**Affiliations:** a Key Laboratory of Phytochemistry and Natural Medicines, Dalian Institute of Chemical Physics, Chinese Academy of Sciences, Dalian 116023, China; b Jiangxi Provincial Key Laboratory for Pharmacodynamic Material Basis of Traditional Chinese Medicine, Ganjiang Chinese Medicine Innovation Center, Nanchang 330000, China; c School of Pharmacy, Shanghai Jiao Tong University, Shanghai 200240, China; d University of Chinese Academy of Sciences, Beijing 101408, China

**Keywords:** Analgesic, Dual-target, mu opioid receptor, M3 receptor, Traditional Chinese medicine, Alkaloid

## Abstract

**Introduction:**

Opioids are the most common antinociceptive drugs, but long-term administration causes serious adverse side effects. *Gelsemium elegans* Benth. is traditionally used as an analgesic agent and mainly contains indole alkaloids with structures different from those in common opioids, indicating distinct pharmacological properties. This work aims to find a new analgesic from *Gelsemium elegans* Benth. and evaluate it *in vitro* and *in vivo*.

**Methods:**

Dihydrokoumine was purified from *Gelsemium elegans* Benth. Binding to mu opioid receptor (MOR), M3 receptor (M3R) and other 15 G protein-coupled receptors were evaluated *in vitro* combined with molecular docking analysis. Analgesic efficacy and side effects were measured *in vivo* using hot-plate, formalin paw, and rotarod tests in mice. Cytotoxicity, acute toxicity in mice and pharmacokinetics were assessed.

**Results:**

A MOR agonist, dihydrokoumine, was first identified from *Gelsemium elegans* Benth. Further investigations showed that dihydrokoumine exhibited selective partial agonist action on the MOR and antagonist action on the M3R among other 15 GPCRs. In *in vivo* mouse models, dihydrokoumine could relieve acute pain and chronic inflammatory pain without drug tolerance and sedative side effects. Additionally, we observed a good safety profile and favorable pharmacokinetic properties.

**Conclusion:**

A MOR partial agonist/M3R antagonist analgesic with reduced side effects was isolated from a traditional Chinese medicine. This study bestows dihydrokoumine as a new dual-target analgesic and as a potential lead compound in long-term pain management.

## Highlights

• A MOR agonist DK was identified from a traditional Chinese medicine.
• DK displayed a selective mixed MOR partial agonist/M3R antagonist profile.
• DK relieves acute pain and chronic inflammatory pain in mouse models.
• DK exhibits a good safety profile and favorable pharmacokinetic properties.
• DK is a new dual-target analgesic without drug tolerance and sedative side effects.

## Graphical abstract

**Figure:**
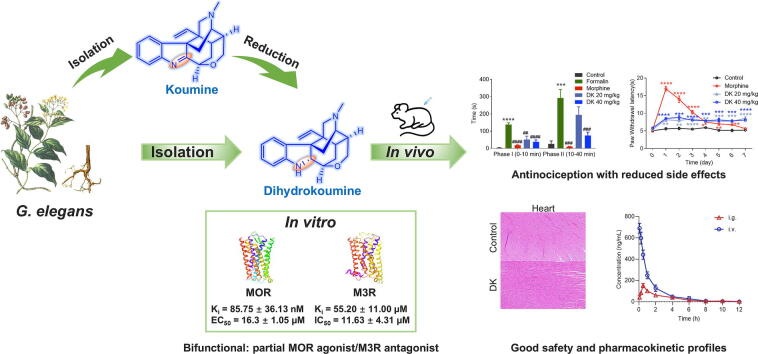

## Introduction

Pain is the symptom of many diseases, often caused by adverse external or internal stimuli, and severe pain degrades quality of life and escalates healthcare costs. Opioid analgesics (i.e., morphine and its derivatives) constitute the favored treatment for managing pain resulting from major trauma, injury, surgical trauma and cancer [1], [2], [3], [4]. However, opioid drugs are plagued by their side effects, including constipation, respiratory depression, vomiting, nausea, addiction and antinociceptive tolerance. Compared with small molecular opioids, opioid peptides usually exhibit largely enhanced analgesic efficacy, but relatively poor receptor selectivity, poor bioavailability *in vivo* and insufficient blood–brain barrier penetration [5], [6], [7]. Therefore, research on new analgesic compounds with good pharmacological and pharmacokinetic properties and reduced side effects is important.

Traditionally, drug design has adhered to the *one target, one drug* principle, to tackle a given disease with high selectivity to avoid undesired side effects caused by off-targets. Consequently, the concept of drugs engaging with multiple targets has historically been viewed unfavorably due to its inherent association with unfavorable side effects. However, with regard to some complex pathologies, such single-target drugs usually fail to achieve favorable therapeutic effects [8], [9], [10]. In contrast, a multiple-target drug may exhibit lower potency for individual targets than a single-target drug but does not necessarily imply diminished efficacy; thus, this type of drug may possess in principle a safer profile [8], [9], [10]. Recent years have witnessed the development of multiple-target drugs. Ding et al. reported a bifunctional MOP/NOP receptor agonist as an effective analgesic without safety and abuse liability concerns [11]. The analgesic tramadol acts as a mu opioid receptor (MOR) agonist and M3 muscarinic acetylcholine receptor (M3R) antagonist [12]; it shows potent antinociception efficacy, less drug addiction and no respiratory depression compared with typical opioids. We have learned that the M3R promotes the endocytosis of the MOR mediated by morphine, and an M3R antagonist is able to relieve opioid withdrawal symptoms and treat opioid addiction [13]. Therefore, developing a compound with MOR agonistic and M3R antagonistic activity is promising for finding a new analgesic lead with high potency and fewer side effects. Additionally, it has been reported that compared with typical potent agonists, partial agonists can improve the side effect profile and safety of opioid agonists [14], [15].

Traditional Chinese medicine is an important resource for the discovery of multi-target drugs. Remarkably, and medical plants rich in alkaloids attracted notable interest, given that alkaloids comprise nearly half of all pharmaceutical natural products. [16], [17]. *Gelsemium elegans* Benth (*G. elegans*) is known for its analgesic effects of alkaloids, which have a unique skeleton, largely differing from the structures of reported MOR agonists [18]. It has been reported that Gelsemium alkaloids exhibit no antinociceptive tolerance like morphine, and their principal component gelsemine induces substantial and selective antinociceptive effects in chronic pain conditions through activation of the spinal α3 glycine/allopregnanolone pathway [18], [19]. To the best of our knowledge, there has not been any research on the effects of Gelsemium alkaloids on opioid receptors.

In this work, we aim to find a potential dual-target analgesic through bioassay-guided purification from *G. elegans* and evaluate its pharmacological activities and related effects.

## Materials and methods

### Reagents

The reagents used to separate alkaloids, including methanol (MeOH) and acetonitrile (ACN), formic acid (FA), ammonium hydroxide, triethylamine, and acetate acid (all HPLC grade), were purchased from Merck KGaA (Darmstadt, Germany). Analytical-grade FA and ammonium hydroxide were obtained from Tianjin Kermel Chemical Reagent Factory (China). Preparative-grade MeOH, ACN and ethanol were purchased from Fulltime (Anhui, China). Industrial-grade ethyl acetate, dichloromethane, ethanol, sodium hydroxide and sulfuric acid were purchased from Chron Chemicals (Chengdu, China). A solution of 0.1 % triethylamine acetate (TEAA) at pH 11 was prepared by mixing acetate and triethylamine in a 1:3 ratio (v/v). Ultra-pure water was produced by a Milli-Q purification system (Millipore, Billerica, MA, US).

Dimethyl sulfoxide (DMSO) was purchased from BIO BASIC INC (Toronto, Canada). Probes Endomorphin-1 and carbachol were purchased from TCI (Japan); [^3^H] DAMGO and microscint 20 cocktail were form PerkinElmer (USA); loperamide, SNC162, MCOPPB and ICI199441 were from Tocris Biosciences (Ellisville, USA); 5-hydroxytryptamine, L-Noradrenaline, isoprenaline were from Amquar (Shanghai, China); DAMGO, 4-DAMP, [^3^H] NMS, Tris HCl (prepare 1 M stock and adjust pH to 7.4), 1 M MgCl_2_, poly ethyleneimine, CP 55940, histamine, zaprinast and dopamine were from Sigma-Aldrich (St Louis, USA). Dulbecco's modified Eagle medium (DMEM), Ham's/F^2^ K medium, Opti-MEM medium, McCoy's 5 A medium, HEPES, Hank's balanced salt solution (HBSS) and fetal bovine serum (FBS) were obtained from Thermo Fisher Scientific (Grand Island, USA). G-418 (Cat. No. FG401) was obtained from TransGen Biotech (Beijing, China). Hydrogenated castor oil used in animal experiments was obtained from Aladin (Shanghai, China).

### Cells

HEK293T,A431,CHO and HUVEC cell lines were obtained from the Type Culture Collection of Chinese Academy of Sciences (Shanghai, China). HEK293-NOP and HEK293T-Mu cells were kindly donated by Professor Olivier Civelli from University of California (Irvine, USA). HEK293-M3, HEK293-Delta, HEK293-Kappa, HEK293-D1, HEK293-D2, HEK293-5-HT2A, HEK293-α2A, CHO-hGPR35 and CHO-CB1 cells were obtained from transfection of corresponding cells as reported before [20]. All GPCRs mentioned above were human GPCRs.

### Animals

Animal use procedures were conducted in compliance with the National Institutes of Health Guide for the Care and Use of Laboratory Animals and approved by the Biological Research Ethics Committee of Shanghai Jiao Tong university (A2017071). Six-week-old C57BL/6 mice and SD male rats were housed free for food and water during acclimation in the animal experiment center for a week.

### Purification, identification, and synthesis of DK

The tuber of *G. elegans* (198 kg) was gathered from Guangxi province and validated by the Institute of Medication at Xiyuan Hospital, China Academy of Traditional Chinese Medicine. *G. elegans* tubers underwent extraction using a solution of 70 % ethanol in water (v/v), and its alkaloid components (486 g) were concentrated using acid-base extraction. The enriched alkaloidal extract (406 g) was then purified using a preparative three-dimensional (3D) high-performance liquid chromatography (HPLC) method, and the first- and second-dimension preparation were presented previously [21]. Six fractions (Fr.1 ∼ Fr.6) were obtained after the first-dimension preparation, and the active fraction Fr.3 was further fractionated into ten fractions (Fr.3–1 ∼ Fr.3–10) in the second-dimension preparation. The third-dimension preparation of the active Fr.3–9 was conducted using a C18CE column (10 mm × 250 mm, 10 μm, Acchrom, China). The mobile phase was composed of (A) H_2_O and (B) ACN both containing 0.1 % TEAA (HAc: TEA = 1:3, pH 11). The elution process employed a gradient of 30–50 % B over 0–30 min. The detection wavelength was 265 nm, the flow rate was 3 mL/min, and the column temperature was held at 30°C. Injection volume totaled 50 μL, and the procedure was completed in 15 cycles. The active fraction Fr.3–9-11 was pooled, and further purified on semi-preparative XCharge C18 column employing an isocratic elution of 10 % methanol. 25 mg of the active compound with a purity higher than 90 % was obtained.

The structures of the purified compounds were determined through HRMS and NMR analysis. Mass spectra were acquired employing the Agilent 1290 Infinity UPLC coupled with the 6540 UHD Q-TOF system (Agilent Technologies, CA, USA), and NMR spectra were obtained on Bruker AV600 spectrometer.

We added 0.6 g koumine (2 mmol, 1.0) to 20 mL tetrahydrofuran and stirred to dissolve, and then cooled the solution at 0–4°C in an ice water bath. 0.23 g LiAlH_4_ (6.0 mmol, 3.0) was added to the solution and stirred at room temperature. After stirring for one hour, 6 mL water was used to quenching the reaction, to which 6 mL 20 wt% NaOH and another 6 mL water were subsequently added. Afterward, the reaction solution was filtered to remove the suspended solids and the filtrate extracted with ethyl acetate three times. The combined ethyl acetate phase was washed with saturated NaCl solution and then dried over Na_2_SO_4_, filtered, and evaporated under reduced pressure. The obtained reaction product was subjected to silica gel column chromatography using dichloromethane-methanol (10:1, v/v) as eluent to afford DK (91.7 %) as a colorless amorphous solid.

### Cell culture

HEK293T and A431 cell were cultured in DMEM medium, and CHO and HUVEC cell lines were in Ham’s F12K medium, both of the two mediums were supplemented with 10 % FBS. HEK293T-Mu, HEK293-Delta, HEK293-Kappa, HEK293-NOP, HEK293-M3 and HEK293-5-HT2A cell lines were cultured in DMEM supplemented with 10 % FBS and 300 μg/mL G418; HEK293-D1, HEK293-D2 and HEK293-α2A cell lines in DMEM supplemented with 10 % FBS, 300 μg/mL G418 and 2 μg/mL Blasticidin; CHO-hGPR35 cell line in 90 % Ham’s F12K supplemented with 10 % FBS and 200 μg/mL Zoecin; CHO-CB1 cell line in Ham’s F12K supplemented with 10 % FBS, 300 μg/mL G418 and 2 μg/mL Blasticidin. All the cell lines were maintained at 37°C under 5 % CO_2_.

### Dynamic mass redistribution (DMR) assays

DMR assays were performed using an Epic® BT system (Corning, NY, USA). All used cell lines were directly seeded in Epic 384-well biosensor microplates with a seeding density of 25,000 cells per well. After being cultured for about 22 h, the cells formed a monolayer with a confluency of about 95 % in the cell culture medium. Then the cells were manually washed using assay buffer (1 × HBSS buffer, 10 mM HEPES, pH = 7.4) and maintained in 30  μL of the assay buffer for 1  h. All samples were dissolved in DMSO and diluted freshly with the assay buffer containing 0.1 % BSA.

The DMR assays were conducted as follows. For the DMR agonist assay, a baseline was established over 2 min before introducing analytes at various concentrations; the resulting induced DMR signals were observed for one hour. In the case of the DMR desensitization assay, cells were pretreated with analytes for one hour before introducing a specific agonist at the indicated concentration, and the agonist-induced DMR signals were monitored for one hour. Finally, for the DMR antagonist assay, cells underwent a 1-hour pretreatment with an antagonist before the addition of a specific agonist at the indicated concentration, and subsequent DMR signals were recorded over one hour. For the MOR assays, HEK293T-Mu cells were used, with endomorphin-1 or loperamide serving as agonists and naloxone as the antagonist.

The receptor selectivity was evaluated against a set of G protein-coupled receptors (GPCRs), including opioid receptor (Delta/Kappa/NOP), dopamine receptor (D1/D2), orphan receptor (GPR35), adrenergic receptor (α2A/β2), serotonin receptor (5HT2A), histamine receptor (H1) and cannabinoid receptor (CB1). Following a 1-hour pretreatment with DK, a 2-min baseline was established before introducing probes and monitoring DMR signals for another hour. The cells and probes were as follows: Delta, HEK293-Delta, SNC162; Kappa, HEK293-Kappa, ICI199441; NOP, HEK293-NOP, MCOPPB; D1, HEK293-D1, dopamine; D2, HEK293-D2, dopamine; GPR35, CHO-K1-hGPR35, zaprinast; 5HT2A, HEK293-5HT2A, 5-hydroxytryptamine; α2A, HEK293-α2A, L-Noradrenaline; β2, A431, isoprenaline; H1, A431, histamine; CB1, CHO-K1-CB1, CP 55940. The other detailed process was the same as above.

### Ca^2+^ response monitored by fluorometric imaging plate reader (FLIPR) assays

FLIPR assay was carried out as reported earlier [22]. Cell lines were seeded in 96-well plates with black walls (Greiner, USA) at 8.0 × 10^4^ cells per well. After approximately 22 h of incubation, the cells were exposed to Calcium-6 dye (Molecular Devices, USA) in HBSS buffer supplemented with 20 mM HEPES (pH 7.4) at 37°C for one hour. Samples, redissolved in DMSO and housed in 96-well drug plates, underwent dilution with FLIPR buffer and subsequent automated introduction to the cells. For FLIPR agonist assessments, changes in intracellular Ca^2+^ concentration were observed at 520 nm with an excitation wavelength of 488 nm for 4 min. In FLIPR antagonist experiments, cells were pre-incubated with the compound for 10 min prior to introducing a specified concentration of agonist. Results were presented as fluorescence (arbitrary units) plotted against time.

### Radioligand binding assays

Radioligand binding assays were conducted using MicroBeta2 (PerkinElmer, USA). Membrane protein preparations MOR and M3R were each prepared at a concentration of 2.5 µg/well. The radio ligands [^3^H] DAMGO (specific for MOR) and [^3^H]-NMS (specific for M3R) were prepared at concentrations of 0.5 nM and 0.2 nM, respectively. DK and the reference compounds (endomorphin-1 for MOR and scopolamine for M3R) were initially dissolved at concentrations of 200 µM and 1 µM, respectively, and then serially diluted in DMSO to create an 8-point, 4-fold serial dilution in triplicate on a 96-well plate. For each well, 1 µL of the serially diluted test compounds was transferred, followed by the addition of 100 µL of membrane stock solution and 100 µL of radioligand. The plates were sealed and shaken at 300 rpm at room temperature for one hour. Upon completion of the binding assay, the contents of each well were filtered through Unifilter-96 GF/C filter plates (PerkinElmer, USA). The filter plates were dried for one hour at 50°C and sealed. The trapped ^3^H (tritium) on the filters was then quantified using the MicroBeta2. The percentage of inhibition or binding was subsequently calculated.

### Docking studies and molecular dynamic (MD) simulations

The active murine MOR’s crystal structure complexed with BU72 (PDB id: 5C1M) [23] and inactive murine M3R bound to tiotropium (PDB id: 4DAJ) [24] were prepared for molecular docking of DK. The molecular docking of compounds against the MOR and M3R model were performed using the Schrödinger software. The molecular mechanics with generalized Born surface area (MM-GBSA) binding free energies of both DK complexed with MOR and M3R were computed using the Schrödinger Prime protein-structure prediction program (Schrödinger Release 2021–01) and docking score was given.

The MD simulation study was performed for 100 ns for the top-ranking docking poses of protein–ligand complexes using the DESOOND module (Schrödinger Release 2022–01), to examine ligand stability within the ligand-receptor complex's binding site. Employing the Desmond system builder, a 10 Å buffered orthorhombic system was established with periodic boundary conditions, encompassing the SPC water model and a DPPC lipid membrane. The OPLS4 force field initiated the process, followed by the addition of Na^+^ and Cl^–^ for neutralization. The trajectory covered 20.0 ps with 10,000 frames, employing an NPT ensemble at 300 K and 1.01325 bar. RMSF (root mean square fluctuation) and RMSD (root mean square deviation) plots were generated along with the MD simulation.

### Cytotoxicity assay

HEK293T, CHO, and HUVEC cell lines were cultured overnight in 96-well plates before exposure to compounds for 24 h. Cell viability was evaluated using the Cell Counting Kit-8 (CCK-8, Beyotime Biotechnology, China), and absorbance at 450 nm was measured with a microplate reader. Cell survival rates were calculated relative to control conditions.

### Hot-plate assay

The hot-plate test in mice was conducted following previously established method [25]. Six-week-old C57BL/6 male mice were used in hot-plate assay (n = 6). Hot-plate response latency (55°C) after drug treatment was used to measure antinociceptive effect, where response was defined by the animal either by licking paws, flicking paws or jumping. Mice were exposed to the hot plate (Ugo Basile model 7280, Comerio, Italy) for a maximum of 20 s to avoid tissue damage. After three days’ baseline measure, mice were injected intraperitoneally (5 mL/kg) with the solvent mixture, morphine (5 mg/kg) or DK (20, 40 mg/kg) and latency was tested 0.5-, 1-, 2-, 3- and 4- hours post-administration. Evaluation of MOR agonistic and M3R antagonistic activity of DK, naloxone (2 mg/kg) and oxotremorine (0.4 mg/kg) were first injected intraperitoneally, immediately followed by the injection of morphine (5 mg/kg, evaluation of MOR) or DK (20 mg/kg).

Subsequent tolerance assessment was conducted through a repeated-injection protocol employing the hot-plate assay. Mice were injected intraperitoneally (5 mL/kg) with the solvent mixture, morphine (5 mg/kg) or DK (20, 40 mg/kg) daily for a continuous seven-day period. The diminishment of the antinociception of drugs in the assay was employed to evaluate the tolerance, with latency measured one hour after drug administration throughout the week.

### Formalin paw test

The formalin test in mice was conducted following established procedures [26]. Six-week-old C57BL/6 male mice were used in hot-plate assay (n = 6). Mice were individually acclimated in a 4-liter glass beaker for 30 min prior to the test. Solvent mixture, formalin (5 %), morphine (5 mg/kg), or DK (20, 40 mg/kg) were injected intraperitoneally (5 mL/kg) 15 min prior to formalin injection. Formalin solution (5 %, 25 μL) was injected into the dorsal surface of the right hind paw using a 50 μL Hamilton syringe with a 30-gauge needle. Following formalin injection, the frequency of paw licking was observed at 5 min-internals in two phases: 0–10 min (phase I) and 10–40 min (phase II). Evaluation of MOR agonistic activity of dihydrokoumine, naloxone (2 mg/kg) was first injected intraperitoneally, immediately followed by the injection of morphine (5 mg/kg) or DK (20 mg/kg).

### Rotarod test.\

Motor coordination were evaluated by the rotarod test, which was conducted as previously reported [26]. Six-week-old C57BL/6 male mice were used in hot-plate assay (n = 6). Mice underwent a three-day training period, twice daily (9:00 am and 3:00 pm), to make them stay balanced on the rotarod apparatus (Columbus), with a 3 cm diameter and rotating at a constant 11 rpm. Motor coordination was evaluated through mice's endurance on the rotating rod. Selection criteria included mice maintaining balance for 60–120 s. On the fourth day, mice were intraperitoneally injected (5 mL/kg) with a solvent mixture, morphine (5 mg/kg), or DK (20, 40 mg/kg) one hour before testing. Latency to fall (in seconds) was recorded after placing mice on the rotarod, with a 120-second cut-off time.

### Acute toxicity studies

Sixteen C57BL/6 mice (25 ± 1 g, 8 males, 8 females) were used in acute toxicity studies. The mice were randomly divided into 2 groups (half male, half female each group) and treated with either solvent mixture (control) or 200 mg/kg DK (DK-treated) daily for 14 days. Throughout the study, the animals were monitored for motor activity, mortality, and body weight changes, which were recorded every other day.

At the end of the experiment (24 h after final DK administration), blood samples were collected via direct heart puncture, allowed to clot at room temperature, and centrifuged at 3000 rpm for 15 min. Serum was separated and stored at 20°C for biochemical analysis. Liver and renal injury markers, including aspartate transaminase (AST), alanine transaminase (ALT), UREA, CREA, albumin (ALB), urine acid (UA) and Glucose (Glu), were measured using an automatic biochemical analyzer (Chemray 240, 420 and 800, Rayto, China).

Following blood collection, the mice were sacrificed, and the heart, liver, spleen, lung and kidney were excised. These tissues were fixed in 4 % paraformaldehyde (PFA), dehydrated gradually using ethanol (50–100 %), and embedded in paraffin after clearing with xylene. Sections (4–5 mm thick) were stained with hematoxylin and eosin (HE) for examination.

### Pharmacokinetics study of DK

Oral bioavailability evaluation was as follows. SD male rats (about 250 g) were used in bioavailability evaluation (n = 3). Mice were given a single dose of DK (20 mg/kg, 5 mL/kg, i.p.) through intragastric administration or tail vein injection. Blood (300 μL) was collected at 0.083, 0.167, 0.25, 0.5, 1, 2, 4, 6, 8, and 10 h, into EDTA tubes. Plasma was obtained by mixing with acetonitrile and centrifugation (3000 r/min, 10 min).

Blood‐brain barrier permeability evaluation was as follows. C57BL/6 male mice (about 25 g) were used in evaluation of blood‐brain barrier permeability (n = 3). Mice were given a single dose of DK (20 mg/kg, 5 mL/kg, i.p.) through intraperitoneal injection. Blood (300 μL) was collected at 0.167, 0.5, 1, 2, 3 and 4 h into EDTA tubes. Plasma was obtained by mixing with acetonitrile and centrifugation (3000 r/min, 10 min). Meanwhile, brains were excised after saline perfusion at these time points. Brain samples were grinded (70 Hz, 90 s) and sonicated (30 s) to break up the tissue, followed by centrifugation (15,000 r/min, 10 min, 80°C) to obtain brain sample.

Brain and plasma drug levels were measured by LC-30AD (Shimadzu Corporation Kyoto, Japan) and SCIEX QTRAP-6500 (AB Sciex, Foster City, CA, USA) and calculated by comparing with internal standard (koumine, 200 ng/mL). The used column was Waters ACQUITY UPLC BEH C18 (2.1*150 mm, 1.7 μm); the mobile phase was composed of (A) 0.1 % FA in water and (B) 0.1 % FA in ACN; the elution gradient was 20 % B (0–3 min), 20–40 % B (3–5 min), 40–95 % B (5–6 min); the flow rate was 0.3 mL/min; and detection was in the positive mode.

### Data analysis

DMR data were acquired using Epic Imager software (Corning, NY, USA) and subsequently processed utilizing Imager Beta 3.7 (Corning, USA), GraphPad Prism 8.0.2 (GraphPad Software Inc., San Diego, CA, US) and Microsoft Excel 2010. Background subtraction was applied to all DMR signals. Concentration-DMR response curves were subjected to nonlinear regression to derive EC50 or IC50 values. Data were expressed as the means ± SD based on three independent experiments.

### Quantification and statistical analysis

Statistical analysis was conducted through GraphPad Prism 8.0.2 and unpaired *t*-test of variance was used to evaluate statistical significance. The level of statistical significance is expressed as a p-value, P < 0.05 was considered as significant difference (*P < 0.05, **P < 0.01, ***P < 0.001, ****P < 0.0001 vs. the control group. #P < 0.05, ##P < 0.01, ###P < 0.001, ####P < 0.0001 vs. the model group).

## Results

### Discovery, purification, and identification of dihydrokoumine, an alkaloid from Gelsemium elegans Benth

**Fig. 1:**
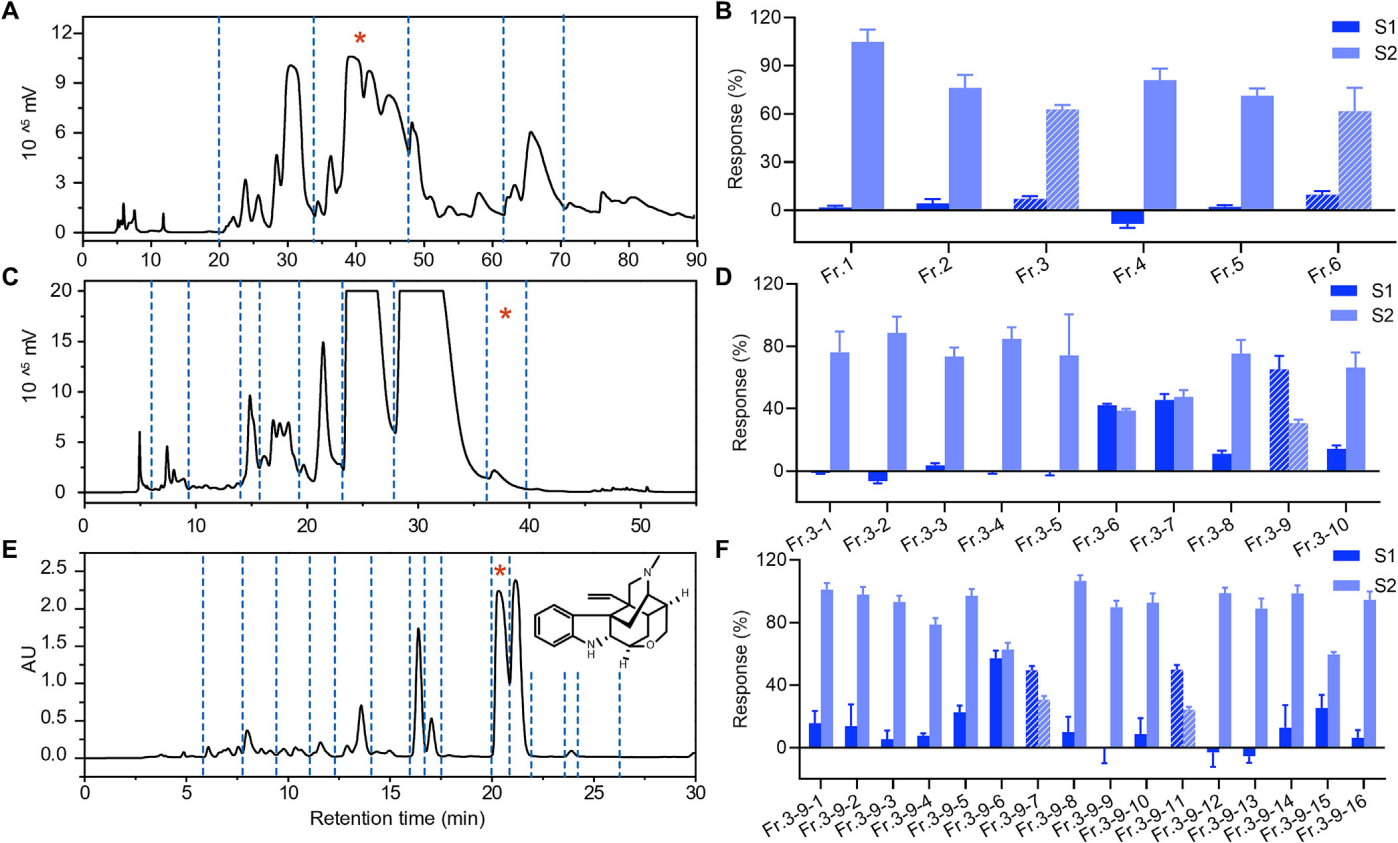
Bioassay-guided purification of MOR agonist from G. elegans alkaloidal extract. (A) The first-dimension preparation of *G. elegans* alkaloidal extract. (C) The second-dimension preparation of Fr. 3. (E) The third-dimension preparation of Fr. 3–9 (structure of DK inserted). (B, D and F) Screening of the first-dimension (B), the second-dimension (D), and the third-dimension (F) fractions in HEK293T-Mu cells. S1: DMR response (percentage) of the fractions to agonist, S2: DMR response (percentage) of 400 nM loperamide (the first- and second- dimension fractions) and 300 nM endomorphin-1 (the third-dimension fractions) with the pretreatment of the fractions for one hour, compared to agonist without pretreatment. For B, D and F, data are presented as mean ± SD (n = 3).

Gelsemium alkaloids are known for their analgesic properties [18]; thus, the alkaloid components were enriched from *G. elegans* for further research. To discover antinociceptive substances from the enriched crude alkaloids, bioassay-guided purification was performed using a combination of the previously developed preparative 3D HPLC method [21] and DMR assay (Fig. 1). The crude alkaloids were then fractioned into six fractions in the first dimension (Fig. 1**A**), and these fractions were tested on HEK293T-Mu cells (Fig. 1**B**). Activity profiling employed a two-step DMR assay: the initial step assessed agonistic activity of each fraction, followed by evaluating their capacity to inhibit the DMR signal triggered by MOR activation via the agonists loperamide or endomorphin-1. Positive DMR signals in the first step (S1), along with reduced loperamide/endomorphin-1 signals (100 % without desensitization) in the second step (S2), suggest potential agonistic activity. For example, Fr. 3 and Fr. 6 induced a small DMR signal in HEK293T-Mu cells and caused an approximate 40 % decrease in the DMR signal of loperamide (400 nM) (Fig. 1**B**), indicating that both Fr. 3 and Fr. 6 contain MOR agonists. Although fractions Fr. 3 and Fr. 6 exhibit comparable agonistic activity, Fr. 3 has a simpler chemical composition (Fig. S1). Consequently, Fr. 3 was selected for further purification to obtain compounds. In the second dimension, ten fractions (Fr. 3–1 ∼ Fr. 3–11) were obtained and then tested for their ability to activate MOR (Fig. 1**C,D**). The DMR response of Fr. 3–9 was elevated nearly 10-fold relative to Fr. 3 (Fig. 1**D**), suggesting that active components were effectively enriched through the second-dimension separation. The third-dimension purification of Fr. 3–9 gave sixteen fractions (Fr. 3–9-1 ∼ Fr. 3–9-16), and Fr. 3–9-7 and Fr. 3–9-11 showed MOR agonist activity (Fig. 1**E,F**). Only one main peak was observed in Fr. 3–9-11, while Fr. 3–9-7 was composed of multiple minor constituents (Fig. 1**E**). Therefore, active Fr. 3–9-11 was chosen for further purification in this work.

After removal of buffer salts, its active component with a purity of 95 % (Fig. S2**A**) was successfully obtained, and its structure was determined by HRMS and NMR (Fig. S2). The compound was determined to be dihydrokoumine (DK) (chemical structure shown in Fig. 1**E,S2A**).

**Fig. 2:**
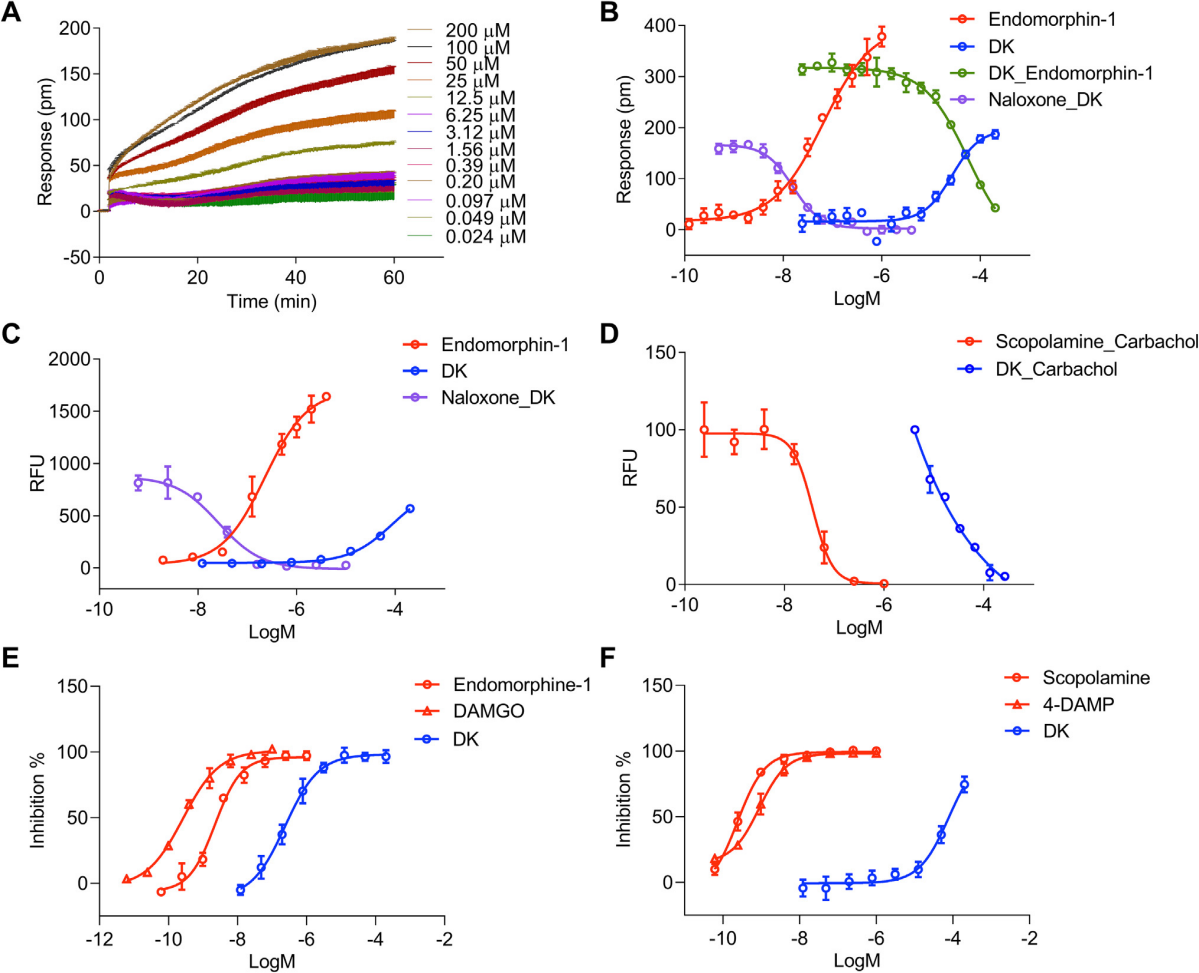
DMR assay, FLIPR assay and radioligand binding assay of DK. (A) Real-time DMR response of DK with different concentrations in HEK293T-Mu cells. (B) Compounds in HEK293T-Mu cells using DMR assay, concentration–response curves of endomorphin-1 and DK; 100 nM endomorphin-1-induced DMR response after DK pretreatment for one hour; 100 μM DK-induced DMR response after naloxone pretreatment for one hour. (C) Compounds in HEK293T-Mu cells using FLIPR assay, concentration–response curves of endomorphin-1 and DK; 100 μM DK-induced Ca^2+^ response after naloxone pretreatment for one hour. (D) Compounds in HEK293-M3 cells using FLIPR assay, the percentage of carbachol-induced Ca^2+^ response after DK (5 nM carbachol) and scopolamine (20 nM carbachol) pretreatment for one hour. (E) Compounds in HEK293 cells using radioligand binding assay, radio ligand [^3^H] DAMGO at a concentration of 0.5 nM, the concentration-inhibition curve of DK, endomorphine-1 and DAMGO. (F) Compounds in HEK293 cells using radioligand binding assay, radio ligand [^3^H] NMS at a concentration of 0.2 nM, the concentration-inhibition curve of DK, scopolamine and 4-DAMP. Data are presented as mean ± SD (n = 3).

### Pharmacological characterization

After determining the active component of DK, we further measured the potency of the compound using DMR assays on MOR in HEK293T-Mu cells. DK concentration-dependently stimulated the DMR response with an EC_50_ value of 16.3 ± 1.05 μM, and this activity caused desensitization to morphine and could be antagonized by naloxone (Fig. 2**A,B**). No background response was observed in HEK293 cells with treatment of DK (Fig. S3). Additionally, DK just reached half of the maximum DMR response of endomorphin-1 (Fig. 2**B**). To validate the activity of DK, FLIPR assays were also performed. DK similarly induced less than half of the maximum Ca^2+^ response of endomorphin-1 in HEK293T-Mu cells, and the response was significantly decreased by naloxone (Fig. 2**C**). Thus, DK is a partial agonist of MOR. Furthermore, we tested the potential antagonistic activity of DK on the M3R in HEK293-M3 cell lines. DK inhibited the carbachol-induced Ca^2+^ response in a concentration-dependent manner with an IC_50_ value of 11.63 ± 4.31 μM (Fig. 2**D**), implying that DK is an antagonist of the M3R. To further confirm the affinity of DK for the membrane proteins MOR and M3R, we conducted radioligand binding assays. For MOR, DK exhibited a K_i_ value of 85.75 ± 36.13 nM with 100 % efficacy, compared to endomorphin-1, which had a K_i_ of 0.80 ± 0.21 nM and a maximal efficacy of 100 % (Fig. 2**E**). For M3R, DK showed a K_i_ value of 55.20 ± 11.00 μM with 74.6 % efficacy, compared to scopolamine, which had a K_i_ of 0.17 ± 0.05 nM and a maximal efficacy of 100 % (Fig. 2**F**). These results confirmed the dual role of DK as a partial agonist at MOR and an antagonist at M3R.

### Molecular docking studies and MD simulations

**Fig. 3:**
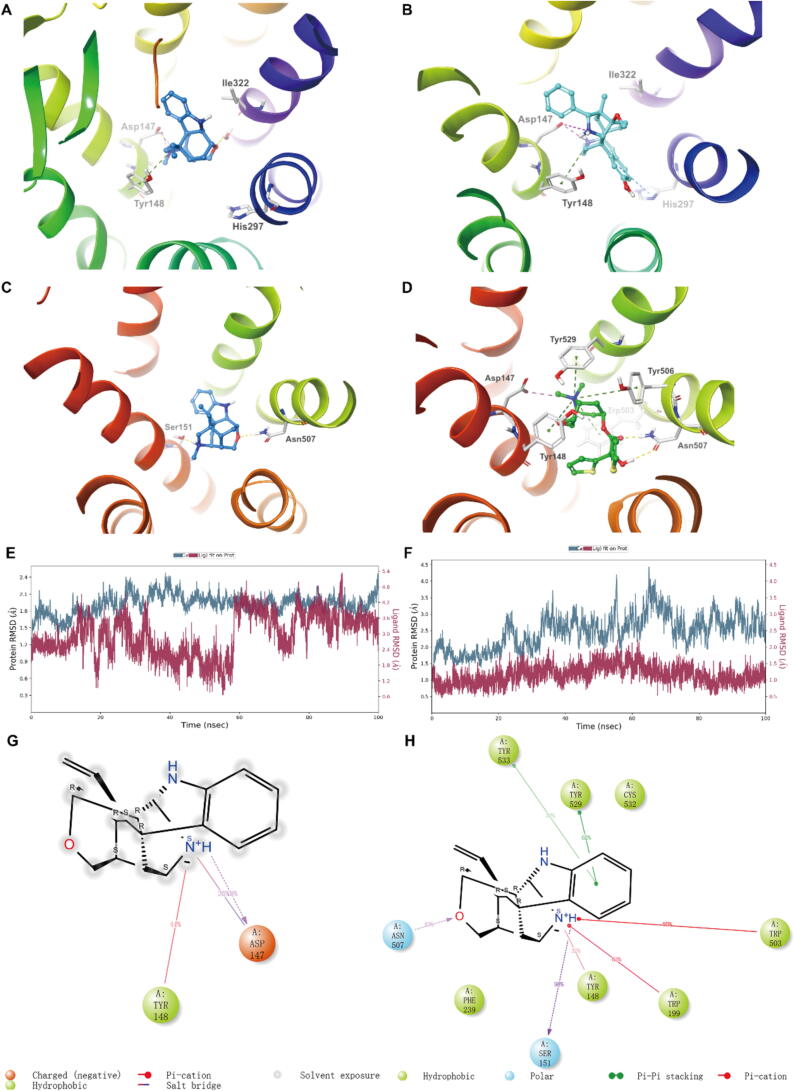
Docking conformation of compounds and receptors and MD simulations (100 ns). (A and C) Predicted binding mode of DK to MOR (A) and M3R (C). (B and D) Reported binding mode of BU72 to MOR (B) and Tiotropium to M3R (D). The yellow dashed lines indicate hydrogen bonds; the purple dashed lines indicate salt bridges; the blue dashed lines indicate π-π bonds; and the green dashed lines indicate π-cation bonds. (E and F) RMSD plots for the docked complex of MOR-DK (E) and M3R-DK (F). (G and H) 2D contacts of protein–ligand complex. MOR-DK (G) and M3R-DK (H).

Specifically, molecular docking of DK to the crystal structures of MOR and M3R was performed. Docking scores of DK complexed with MOR and M3R receptors were −6.65 and −8.70; MM-GBSA free energies of binding of DK to MOR and M3R were −38.20 and −17.97 kcal/mol, respectively. Furthermore, DK interacted with MOR via one salt bridge (Asp147, 1.97 Å) and one Pi-cation (Tyr148, 4.5 Å) (Fig. 3**A**), both of which were also contained in the interactions between BU72 (with another Pi-Pi interaction) and MOR (Fig. 3**B**), indicating that DK and BU72 have similar but not identical docking poses with MOR. Binding modes changed with regard to M3R—two hydrogen bonds (S151, 1.97 Å; N507, 2.05 Å) existed between DK and M3R (Fig. 3**C**), which largely differed from the interactions between tiotropium and M3R (including one hydrogen bond, one salt bridge and four π-cation interactions) (Fig. 3**D**).

The protein–ligand complexes were further subjected to MD simulations for 100 ns to analyze the stability of simulated system through RMSD and RMSF values [27]. The RMSD plots for the protein–ligand complexes of MOR-DK and M3R-DK exhibited fluctuation of less than 3 Å, demonstrating the stability of DK within the binding pockets (Fig. 3**E,F**). The structural integrity and atomic mobility of the complexes were evaluated using RMSF, which showed that most residues fluctuated below 3 Å and a few residues showed fluctuation up to 3.3 Å for MOR-DK complex and 4.0 Å for M3R-DK complex (Fig. S4**A,B**). The favorable interactions between the residues of MOR and DK were found with Asp 147 and Tyr 148 (Fig. 3**G,S4C**), and interactions were found with Tyr 148, Ser 151, Trp 199, Trp 503, Asn 507, Tyr 529 and Tyr 533 with regard to M3R and DK (Fig. 3**H,S4D**).

**Fig. 4:**
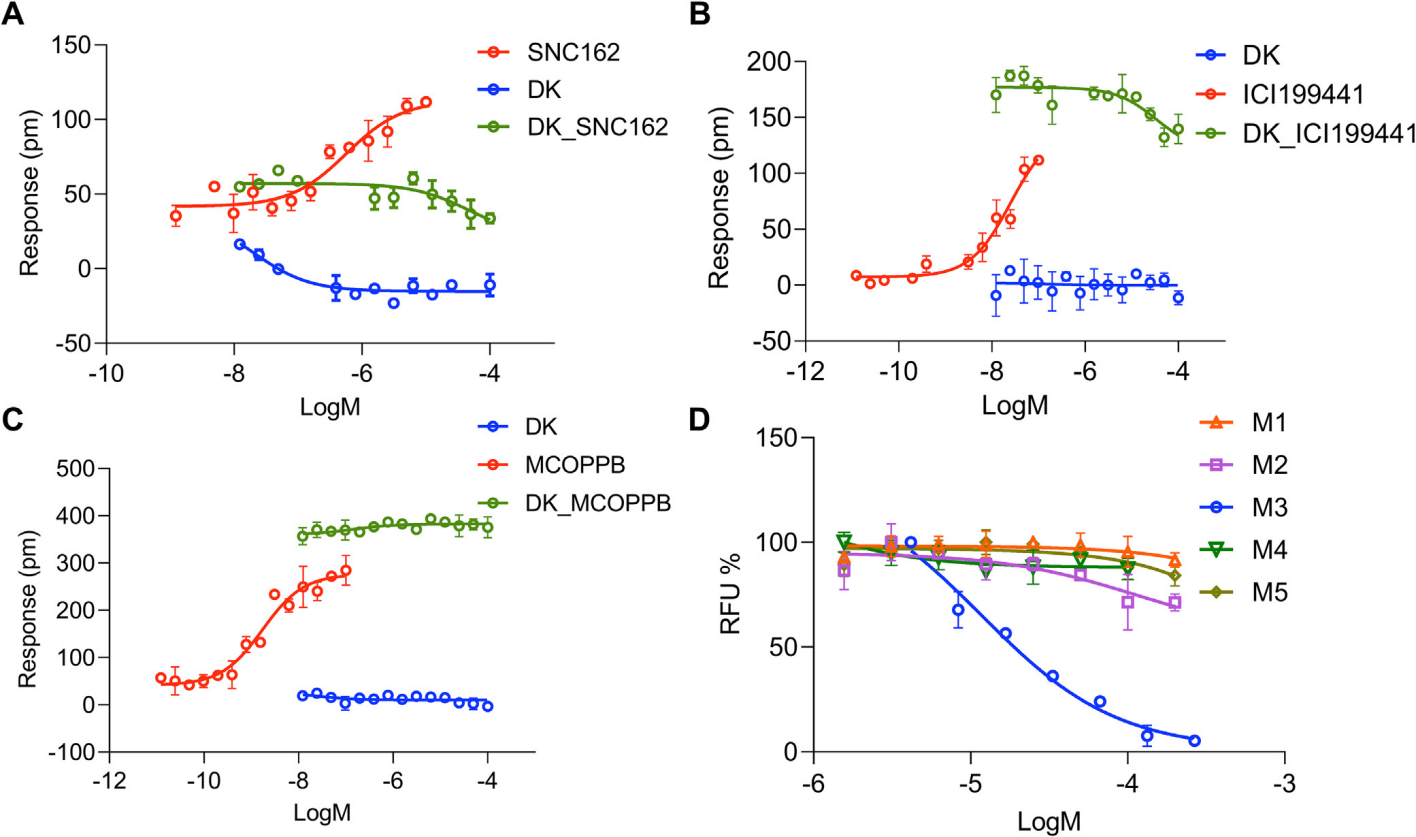
Receptor selectivity investigation of DK. (A-C) DMR assay, compounds in HEK293-Delta (A), HEK293-Kappa (B), and HEK293-NOP (C) cells, concentration–response curves of DK, SNC162, ICI199441 and MCOPPB; 2.5 μM SNC162-, 25 nM ICI199441- and 25 nM MCOPPB-induced DMR response after DK pretreatment for one hour. (D) FLIPR assay, compounds in CHO-M1, HEK293-M2, HEK293-M3, HEK293-M4 and CHO-M5 cells, the percentage of carbachol-induced Ca^2+^ response after DK pretreatment for one hour. Data are presented as mean ± SD (n = 3).

### Evaluation of receptor selectivity of DK on opioid, muscarinic and other GPCR receptors

The Delta/Kappa/NOP opioid activity profile of DK was examined in HEK293-Delta, HEK293-Kappa, and HEK293-NOP cells using DMR agonist and desensitization assays. DK was unable to trigger a concentration-dependent DMR response and had little effect on the DMR response produced by the respective probes (Fig. 4**A-C**), suggesting that DK was selective for MOR against the other three subtypes. In addition, a high-concentration of DK decreased the DMR response of SNC162 and ICI199441 (Fig. 4**A,B**), showing its very weak antagonist effects on Delta and Kappa receptors. Next, muscarinic receptor subtype selectivity (M1R/M2R/M4R/M5R) was evaluated by FLIPR assay, and DK could not concentration-dependently inhibit the Ca^2+^ response induced by the agonist (carbachol) of M1R/M2R/M4R/M5R (Fig. 4**D**). Finally, selectivity among eight other GPCRs was investigated by DMR desensitization assay, including dopamine receptor (D1/D2), orphan receptor (GPR35), serotonin receptor (5-HT2A), adrenergic receptor (α2A/β2), histamine receptor (H1) and cannabinoid receptor (CB1). DK was found almost unable to reduce the response of the corresponding probes on the eight receptors (Fig. S5). Consequently, DK is a highly selective MOR partial agonist/M3R antagonist.

### Semisynthesis of DK and pharmacological activity verification

**Fig. 5:**
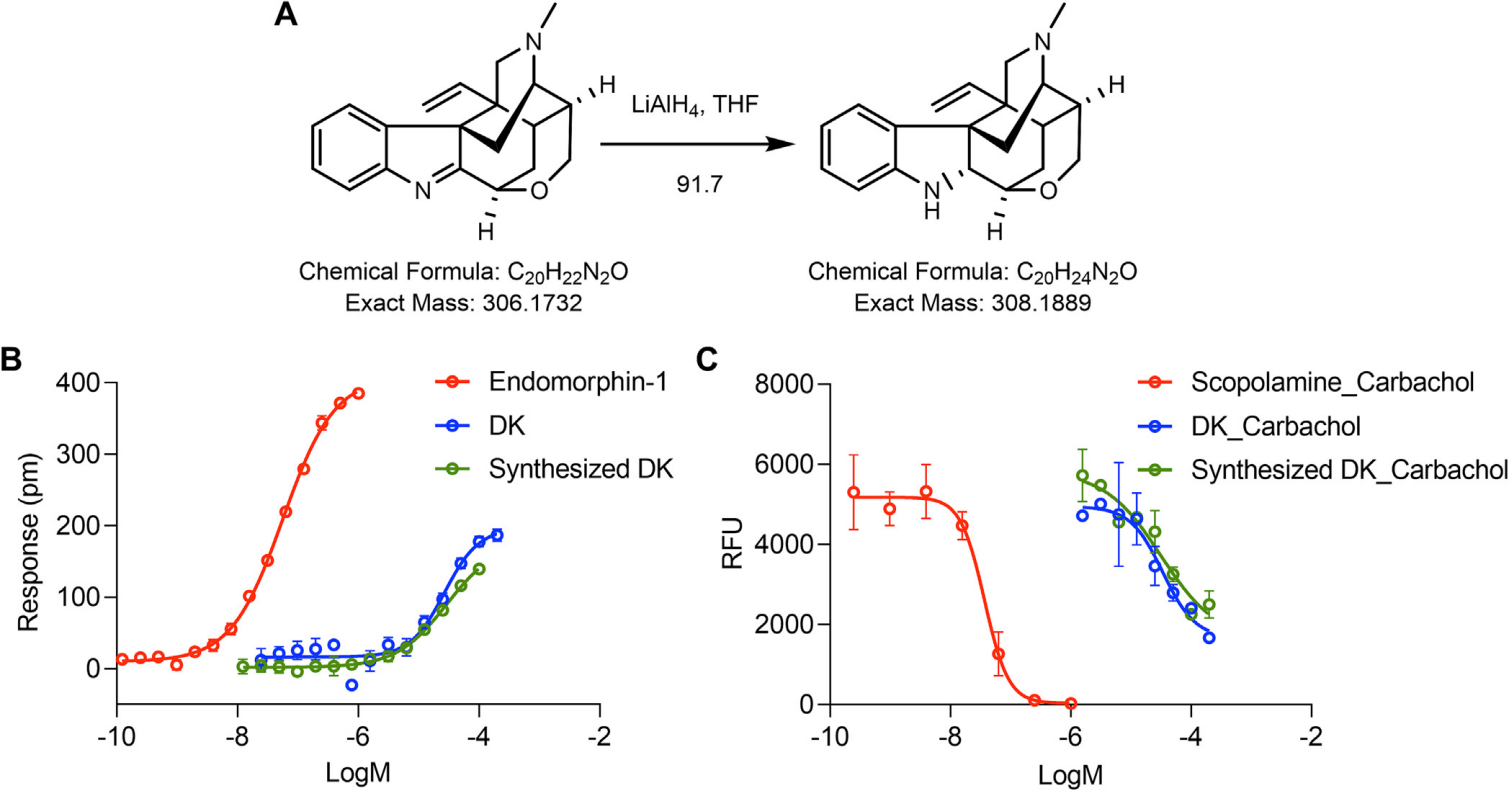
Semisynthesis of DK and activity validation. (A) Synthesis of DK. (B) Concentration-response curves of endomorphin-1, DK and S1 in HEK293T-Mu cells using DMR assay. (C) Compounds in HEK293-M3 cells using the FLIPR assay, carbachol-induced Ca^2+^ responses after scopolamine (5 nM carbachol), DK (20 nM carbachol), and synthesized DK (20 nM carbachol) pretreatment for one hour. Data are presented as mean ± SD (n = 3).

False-positive results might occur in bioassay-guided purification due to complex natural products. We semisynthesized DK to ascertain the activity of isolated DK and provide sufficient amounts for further pharmacological assessment. Koumine, a principal component of *G. elegans*, was obtained in a yield of 29.13 g. It served as the starting material for a selective reduction reaction, using THF as the reaction solvent and LiAlH_4_ as the reductant (Fig. 5**A**). Finally, DK was obtained in 91.7 % yield and purified through preparative HPLC. The semisynthesized compound and the purified DK exhibited identical chromatographic retention patterns and spectral data. Similar to purified DK, semisynthesized DK could generate a concentration-dependent response in MOR and M3R, and its activity was close to that of purified DK (Fig. 5**B,C**). These finding verify that the isolated compound serves as a mixed MOR partial agonist/M3R antagonist.

### Antinociceptive efficacy and side effects of DK

**Fig. 6:**
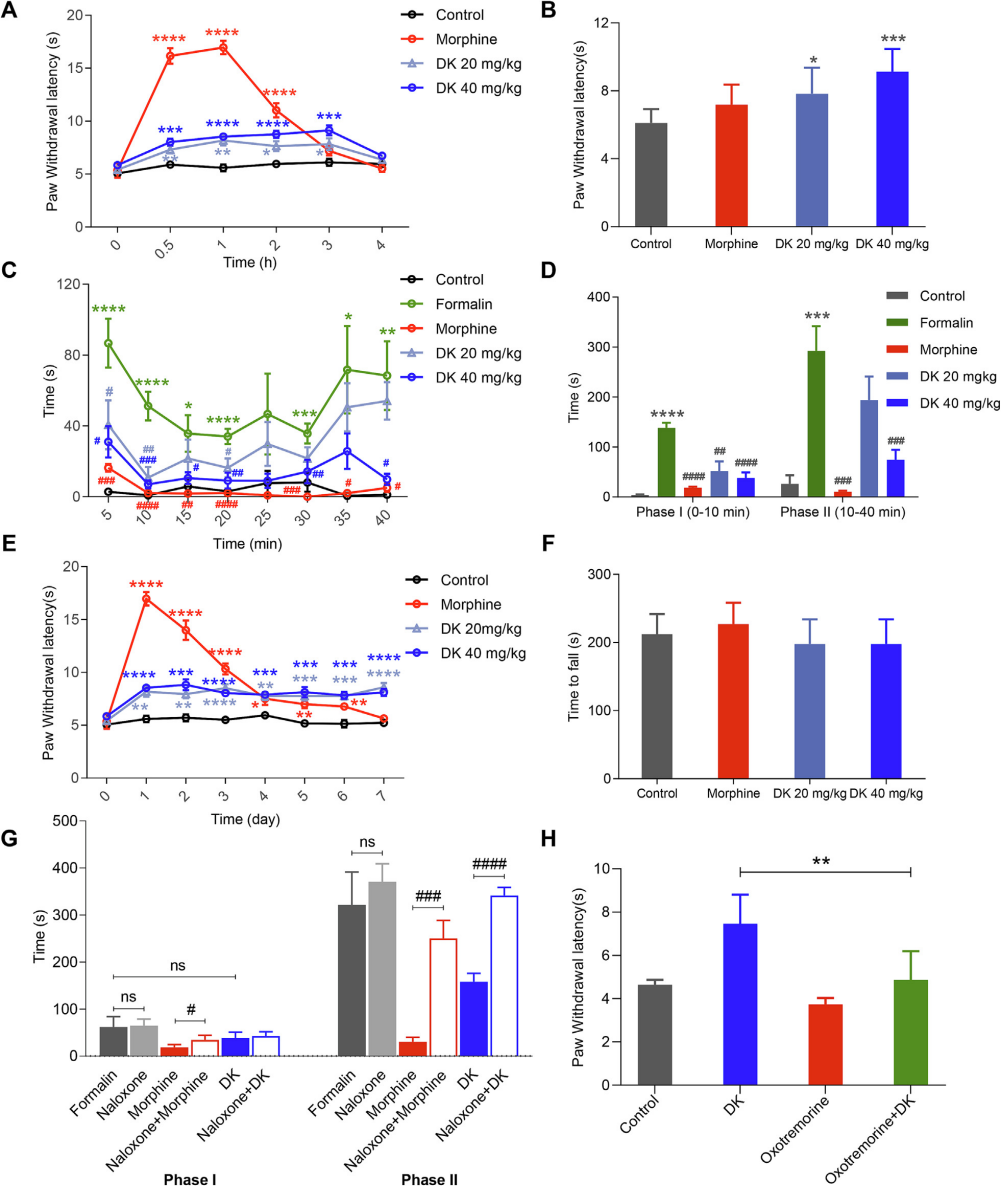
Antinociceptive effects of DK and its side effects. (A and B) Effect of different concentrations of DK on acute pain in the hot plate assay (morphine was 5 mg/kg), time course effect (A) and effect at 3 h (B). (C) Time course effects of DK in the formalin assay (morphine was 5 mg/kg; formalin was 5 %, 20 mL). (D) Cumulative effects in Phase I (0–10 min) and Phase II (10–40 min) of DK (morphine was 5 mg/kg, formalin was 5 %, 20 μL). (E) The tolerance of DK (morphine was 5 mg/kg). (F) The effect of DK on locomotion in the rotarod assay. (G) The cumulative effects of naloxone on antinociception of DK in the formalin test in Phase I (0–10 min) and Phase II (10–40 min) of DK (morphine was 5 mg/kg, DK was 20 mg/kg, naloxone was 2 mg/kg and formalin was 5 %, 20 μL). (H) The cumulative effects of oxotremorine on antinociception of DK in the hot plate assay (oxotremorine was 0.4 mg/kg, DK was 20 mg/kg). Data are presented as mean ± SEM (n = 6–8 mice per group). *P < 0.05, **P < 0.01, ***P < 0.001, ****P < 0.0001 vs. the control group. #P < 0.05, ##P < 0.01, ###P < 0.001, ####P < 0.0001 vs. the model group.

First, the antinociceptive activity of DK was tested in an acute pain model using a hot-plate assay, in which the pain threshold to a thermal stimulus was measured. DK produced potent antinociception in a dose-dependent manner in the hot-plate test (Fig. 6**A**). The antinociception of morphine was largely reduced after one hour and even disappeared at 3 h, while DK’s antinociception was stable and could last at least 3 h (Fig. 6**A,B**). Therefore, DK produces long-lasting antinociceptive effects. A formalin assay was further performed to test DK. In this assay, both acute and persistent inflammatory pain responses were assessed, including two distinct periods, an early phase (Phase I) lasting the first 5 min (corresponding to acute neurogenic pain) and a later phase (Phase II) lasting from 10-50 min (corresponding to inflammatory pain) after injection of formalin [28]. Consequently, DK dose-dependently caused a remarkable decrease in the licking time in Phase I at both doses and in Phase II at high doses (Fig. 6**C,D**), indicating that DK worked both in acute and persistent inflammatory pain.

Opioids usually have many unfavorable side effects, such as antinociceptive tolerance, respiratory depression and constipation. Antinociceptive tolerance of DK was evaluated through daily administrations over 7 days in the hot-plate assay (Fig. 6**E**). Compared with morphine (5 mg/kg), no antinociceptive tolerance was observed with DK (20 and 40 mg/kg). Since strong sedation might affect the results of antinociception studies and most analgesics bear sedative effects, we tested DK for potential sedative effects by observing its influence on locomotion in a rotarod assay. DK was found not a sedative at the tested doses (Fig. 6**F**). Taken together, DK exhibited antinociceptive efficacy on both acute and persistent inflammatory pain without antinociceptive tolerance and sedation side effects.

Furthermore, we investigated whether the antinociception of DK worked through MOR or M3R. Naloxone antagonized the antinociception of DK in formalin test (Fig. 6**G**) and oxotremorine markedly diminished the antinociceptive efficacy of DK (Fig. 6**H**). It is indicated that the antinociceptive effect of DK worked through both MOR and M3R. Interestingly, naloxone failed to decrease the pain threshold of DK in hot plate assay (Fig. S6), largely due to partial agonism of DK on MOR, which may explain its less potent antinociceptive effect compared to full MOR agonists.

### Assessment of the safety and pharmacokinetics of DK

**Fig. 7:**
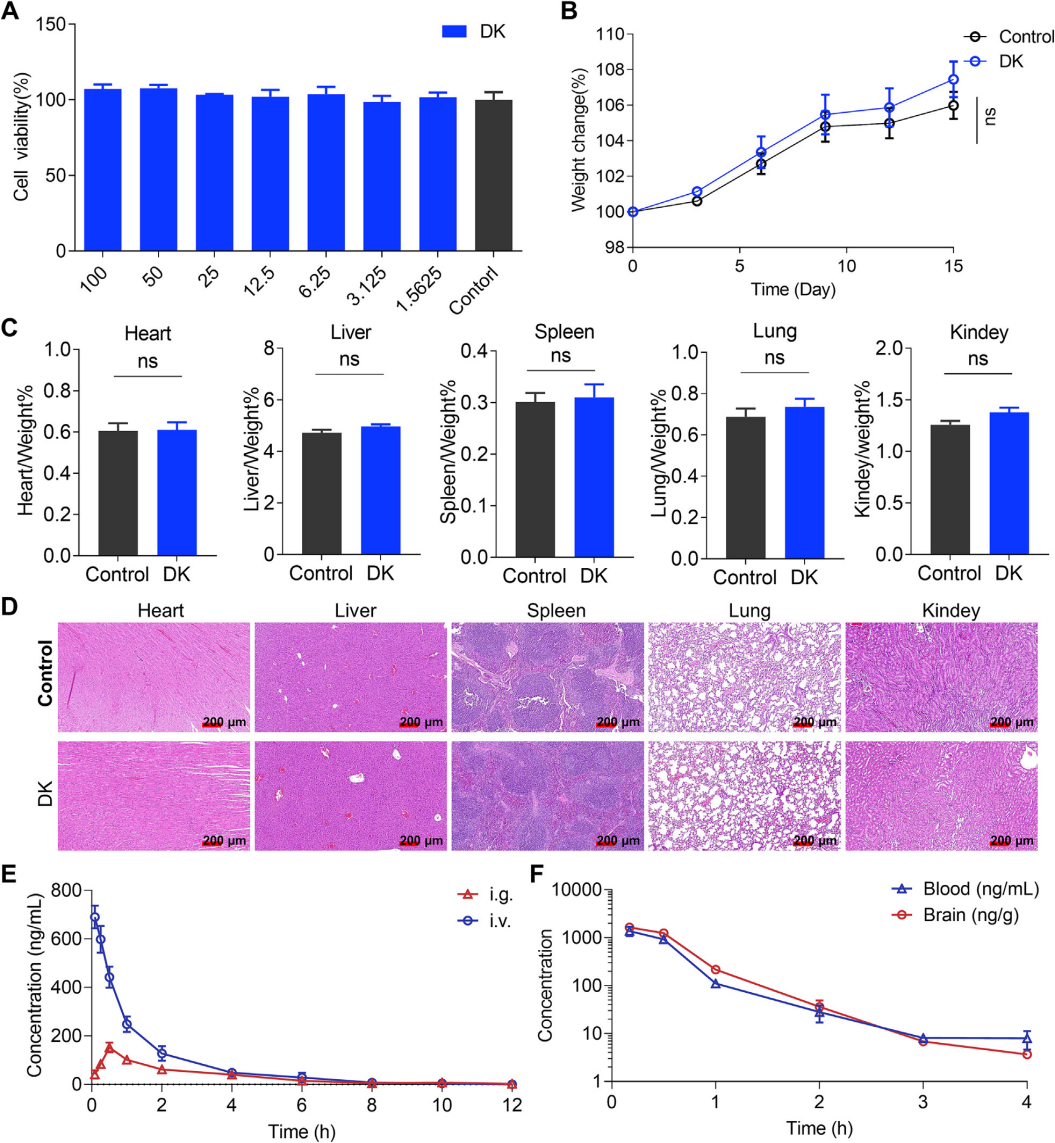
Assessment of the safety and pharmacokinetic properties of DK. (A) Cytotoxicity test of DK in HUVEC cells. (B) Weight-time curve of mice after intraperitoneal injection of DK (200 mg/kg). (C) Main organ coefficients (heart, liver, spleen, lung and kidney) of mice after intraperitoneal injection of DK (200 mg/kg). (D) Histopathological analysis of major organs (heart, liver, spleen, lung, and kidney) in DK-treated mice compared to controls. (E) Concentration-time curve in plasma after intragastric (i.g.) injection and intravenous (i.v.) injection of DK (200 mg/kg) in rats. (F) Concentration-time curve in the brain and plasma after intraperitoneal injections of DK (200 mg/kg) in mice. Data are presented as mean ± SEM (n = 8 mice per group).

Drug safety evaluation was conducted *in vivo* and *in vitro*. The cytotoxicity of DK was first examined in HUVEC, CHO and HEK293T cell lines. DK was noncytotoxic in CHO and HUVEC cells (Fig. 7**A,S7**) and had a little effect on HEK293T cells but not in a concentration-dependent manner (Fig. S7). Assays on semisynthesized DK obtained similar results (Fig. S7). Moreover, the acute toxicity results showed consistent body weight gain in all mice, with no significant differences between DK-treated and control groups (Fig. 7**B**). No visible abnormalities were detected in the major organs, and the organ coefficients for the heart, liver, spleen, lung, and kidney were comparable between the two groups (Fig. 7**C**). Serum biochemical indicators and histological analysis of major organs (via HE staining) revealed no significant structural or biochemical differences (Fig. 7**D,S8**). These results indicated the safety of DK at a dose of 200 mg/kg.

Pharmacokinetic analysis, including oral bioavailability and blood‐brain barrier permeability, was also examined. After intragastric (i.g.) administration, DK remained in the plasma at the highest concentrations at 15 min and then decreased gradually (Fig. 7**D**). and the bioavailability was 42.6 % (details in Supplementary material). Interestingly, the brain concentrations of DK − expressed as area under curve (AUC) − exceed the plasma concentrations in mice models, and Kp (AUC_brain_/AUC_plasma_) reached 1.27 (details in Supplementary material). These findings suggest that DK exhibits favorable pharmacokinetic properties, including effective oral absorption and the ability to permeate the blood–brain barrier.

## Discussion

Pain management remains a challenge, and the most commonly used opioid drugs are plagued by their adverse side effects. The development of multi-target drugs would be promising to obtain a new analgesic with high potency and fewer side effects [8], [9], [10]. Generally, compared with opioid peptides, alkaloid analgesics display better receptor selectivity, degradation performance and blood–brain barrier penetration. Therefore, to find this potential analgesic, we chose a traditional Chinese medicine (*G. elegans*.) rich in alkaloids to carry out further research. We combined the preparative 3D HPLC method [21] and DMR assay to achieve bioassay-guided fractionation from *G. elegans*. Fr. 3–9-11 was found to be active at the MOR and was identified as DK. Further investigations showed that DK demonstrates a more selective pharmacological action, exhibiting partial agonism at the MOR and antagonism at the M3R, without affecting other opioid receptor subtypes (Delta/Kappa/NOP opioid receptors), muscarinic receptor subtypes (M1R/M2R/M4R/M5R), or several other GPCRs. This selective mechanism of action may contribute to a more favorable side effect profile compared to tramadol, which involves broader receptor interactions [12]. To the best of our knowledge, natural compounds with MOR agonistic and M3R antagonistic activities have not yet been reported. Interestingly, DK has a distinct chemical structure compared to existing MOR agonists and M3R antagonists, and the molecular docking analysis highlighted its unique binding modes to both MOR and M3R, implying its distinct pharmacological properties. Additionally, the radioligand binding assay results further confirm DK's dual mechanism and bifunctional activity.

To avoid false-positive results arising from the complexity of natural products and meet the amount required in the pain model *in vivo*, we semisynthesized DK and tested its activity on MOR and M3R again. The synthesized DK was identical to the purified compound in structure and activity, and exhibited antinociception both in thermal-induced acute pain and chemical-induced inflammatory pain. The synthesized DK was found to be effective in a dose-dependent manner. Furthermore, the mixed MOR agonistic/M3R antagonistic activities of DK established *in vitro* could be reinforced by the *in vivo* results of the hot plate assay and formalin assay, and the antinociceptive effect of DK is modulated by both MOR and M3R. Considering the antinociception of DK during both acute and inflammatory phases in the formalin test, it is conceivable that its activity is modulated by direct effects within the central nervous system [29], [30]. Accordingly, our pharmacokinetic analysis indicates that DK is able to penetrate the blood–brain barrier. The side effects of DK were then estimated. Considering that one of the principal side effects of narcotic analgesics is tolerance, antinociceptive tolerance of DK was tested. Continuous DK administration does not lead to tolerance development, highlighting its more favorable profile for long-term use in pain management, with a reduced risk of developing tolerance compared to morphine. Given that most analgesics bear sedation, we assessed DK for potential sedative effects and determined no sedation at antinociceptive doses. These properties suggest that DK could serve as a safer and more sustainable analgesic option compared to traditional opioids like morphine. In summary, DK’s current safety and pharmacokinetic profiles are favorable, though its efficacy could be improved through structure optimization. Enhancing its dual-target interactions based on molecular docking results, along with combinatorial chemistry and structure–activity relationship studies, could lead to a more effective analgesic with an improved therapeutic profile.

## Conclusion

In summary, we identified an alkaloid, DK, from *G. elegans*, and it is a selective bifunctional MOR partial agonist/M3R antagonist among other 15 GPCRs. DK achieved antinociceptive effects on the *in vivo* acute pain and inflammatory pain in mouse model with reduced tolerance and no sedative side effects. A good safety profile and satisfactory pharmacokinetic properties were also observed. These findings bestow DK as a new dual-target analgesic and a promising lead in long-term pain management.

## Compliance with Ethics

All experiments involving animals were conducted according to the National Institutes of Health Guide for the Care and Use of Laboratory Animals and approved by the Biological Research Ethics Committee of Shanghai Jiao Tong university, China (A2017071).

Author Contributions

Conceptualization, Jixia Wang, Yanfang Liu and Xinmiao Liang; Methodology, Jixia Wang and Yan Zhang; Investigation, Dian Liu, Tao Hou, Han Zhou, Yaopeng Zhao, Liangliang Zhou and Cuiyan Cao; Writing − Original Draft, Dian Liu; Writing − Review & Editing, Jixia Wang; Funding Acquisition, Jixia Wang, Dian Liu and Han Zhou; Supervision, Yanfang Liu and Xinmiao Liang.

Ethical statement

All experiments involving animals were conducted according to the National Institutes of Health Guide for the Care and Use of Laboratory Animals and approved by the Biological Research Ethics Committee of Shanghai Jiao Tong university, China (A2017071).

All authors have read this paper and approved to submit it to this journal. There is no conflict of interest of any authors in relation to the submission.

## CRediT authorship contribution statement

**Dian Liu:** Investigation, Writing – original draft, Funding acquisition. **Jixia Wang:** Conceptualization, Methodology, Writing – review & editing, Funding acquisition. **Tao Hou:** Investigation. **Yan Zhang:** Methodology. **Han Zhou:** Investigation, Funding acquisition. **Yaopeng Zhao:** Investigation. **Liangliang Zhou:** Investigation. **Cuiyan Cao:** Investigation. **Yanfang Liu:** Conceptualization, Supervision. **Xinmiao Liang:** Conceptualization, Supervision.

## Funding

This work was supported by Project of National Natural Science Foundation of China (No. 82374021), the innovation program of science and research from DICP, CAS (No. DICP I202211), Jiangxi Provincial Natural Science Foundation (No. 20224ACB216020), Jiangxi Provincial Natural Science Foundation (No. 20232BAB216134), and Jiangxi Provincial Natural Science Foundation (No.20232BAB205021).

## Declaration of competing interest

The authors declare that they have no known competing financial interests or personal relationships that could have appeared to influence the work reported in this paper.
