# Supplementary material for: Dihydrokoumine, a dual-target analgesic with reduced side effects isolated from a traditional Chinese medicine
Source: J Adv Res. 2024 Oct 24;74:637–49. doi: 10.1016/j.jare.2024.10.011 (PMC12302426; doi:10.1016/j.jare.2024.10.011)
Supplement: Supplementary Data 1 [file mmc1.docx]

**Dihydrokoumine, a dual-target analgesic with reduced side effects isolated from a traditional Chinese medicine**

Dian Liu^a,b,1^, Jixia Wang ^a,b,1^, Tao Hou^a,b^, Yan Zhang^c^, Han Zhou^a,b,d^, Yaopeng Zhao^a,b^, Liangliang Zhou^b^, Cuiyan Cao^a,b^, Yanfang Liu^a,b*^, Xinmiao Liang^a,b*^

^a^Key Laboratory of Phytochemistry and Natural Medicines, Dalian Institute of Chemical Physics, Chinese Academy of Sciences, Dalian 116023, China.

^b^Jiangxi Provincial Key Laboratory for Pharmacodynamic Material Basis of Traditional Chinese Medicine, Ganjiang Chinese Medicine Innovation Center, Nanchang 330000, China.

^c^School of Pharmacy, Shanghai Jiao Tong University, Shanghai 200240, China.

**^d^**University of Chinese Academy of Sciences, Beijing 101408, China

^1^These authors contributed equally to this work.

***Corresponding author**

Professor Xinmiao Liang, Key Laboratory of Phytochemistry and Natural Medicines, Dalian Institute of Chemical Physics, Chinese Academy of Sciences, Dalian 116023, China Tel.: +86-411-84379519; fax: +86-411-84379539. E-mail address: [liangxm@dicp.ac.cn](mailto:liangxm@dicp.ac.cn) (X.L.).

*Additional corresponding author:

Professor Yanfang Liu, Key Laboratory of Phytochemistry and Natural Medicines, Dalian Institute of Chemical Physics, Chinese Academy of Sciences, Dalian 116023, China. E-mail address: [liuyanfang@dicp.ac.cn](mailto:liuyanfang@dicp.ac.cn).

Structure Elucidation of DK

Dihydrokoumine, white powder, HR-ESI-MS (*m/z*): 309.1982 [M+H]^+^, calculated for C_20_H_22_N_2_O. ^1^H NMR (400 MHz, MeOD) δ 7.21 (d, *J* = 7.4 Hz, 1H, H-9), 7.03 (td, *J* = 7.7, 1.1 Hz, 1H, H-11), 6.75 (td, *J* = 7.4, 0.8 Hz, 1H, H-10), 6.66 (d, *J* = 7.7 Hz, 1H, H-12), 5.57 (dd, *J* = 18.3, 11.0 Hz, 1H, H-19), 4.63 (dd, *J* = 5.2, 1.0 Hz, 1H, 3), 4.24 (m, 1H, H-18), 4.20 (dd, *J* = 11.9, 5.0 Hz, 1H, H-18β), 3.62 (d, *J* = 11.9 Hz, 1H, H-17), 3.42 (d, *J* = 2.3 Hz, 1H, H-2), 2.75 (overlapped, 2H, H-5, H-21), 2.60 (overlapped, 2H, H-6β, H-16), 2.50 (s, 3H, N-Me), 2.46 (d, *J* = 11.7 Hz, 1H, H-21β), 2.21 (overlapped, 3H, H-14, H-15), 2.06 (dd, *J* = 14.6, 3.9 Hz, 1H, H-6α). ^13^C NMR (151 MHz, MeOD) δ 153.54 (C-13), 142.59 (C-19), 135.12 (C-8), 128.88 (C-11), 124.25 (C-9), 119.84 (C-10), 113.64 (C-18), 111.46 (C-12), 76.94 (C-2), 70.47 (C-3), 62.43 (C-17), 59.10 (C-5), 58.88 (C-21), 47.24 (C-7), 42.83 (C-20), 42.45 (N-Me), 38.53 (C-16), 33.04 (C-6), 30.98 (C-15), 20.87 (C-14). The NMR data were in consistent with those in the literature ^1^.

**Calculation of Bioavailability and Blood-brain Barrier Permeability**

**Bioavailability**

Intragastric administration (AUC)_0-12 h_ = 384 (h•ng•mL^-1^)

Intravenous injection (AUC) _0-12 h_ = 901.9 (h•ng•mL^-1^)

Bioavailability (%) = 384/901.9*100% = 42.6%

**Blood‐brain Barrier Permeability**

In brain (AUC) _0-4 h_ = 933.4 (h•ng•g^-1^)

In plasma (AUC) _0-4 h_ = 733 (h•ng•mL^-1^)

Kp brain (AUC_brain_/AUC_plasma_) = 933.4/733 = 1.27


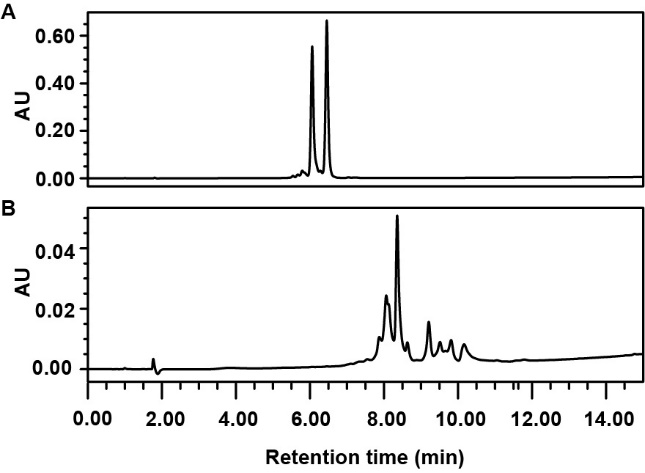


**Fig. S1.** **The reanalysis chromatogram of Fr.3 (A) and Fr.6 (B) on the XCharge C18 column (4.6 mm × 150 mm, 5 μm, Waters).** The mobile phase consists of (**A**) 0.1% formic acid in H_2_O (v/v) and (**B**) 0.1% formic acid in MeOH (v/v), the gradient was 0-50% B (0-15 min), the flow rate was 1 mL/min, the detection wavelength was 254 nm, and the column temperature was 30^o^C.

**
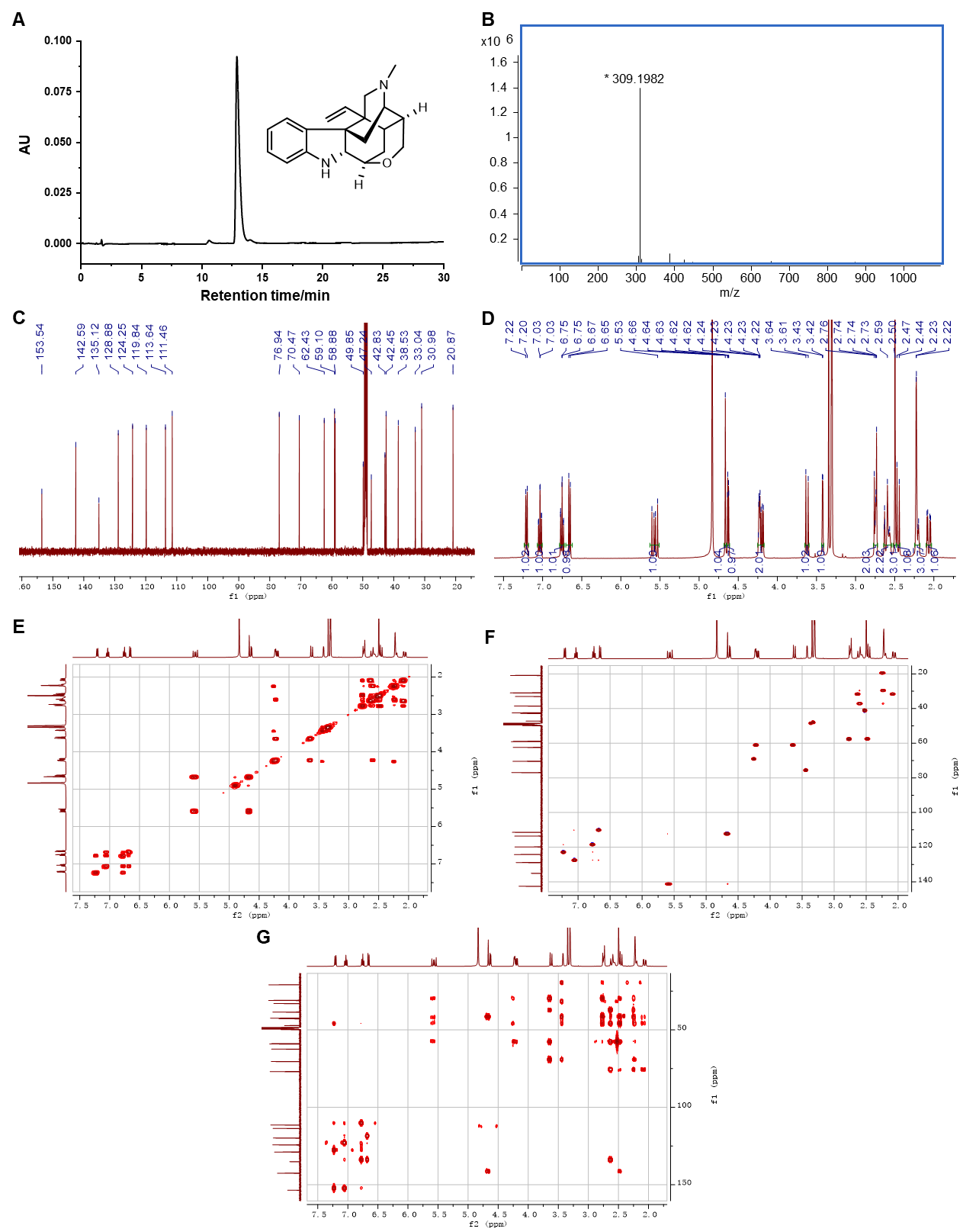
**

**Fig. S2. Structure elucidation of DK.** (**A**) Purity evaluation of DK (chemical structure inserted); (**B**) HRMS spectrum of DK; (**C**)-(**G**) ^13^C NMR, ^1^H NMR, ^1^H-^1^H COSY, HSQC and HMBC spectrums of DK.


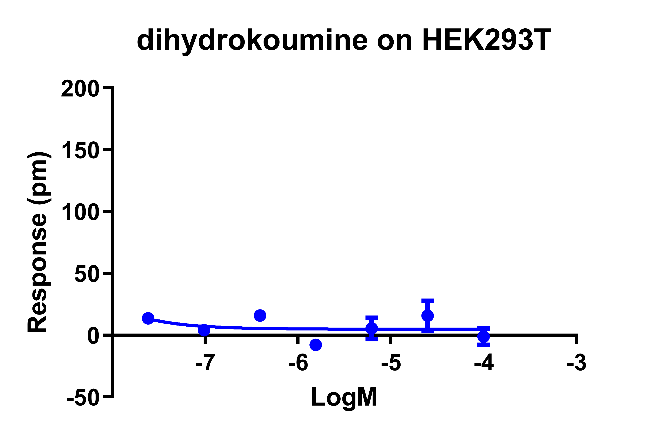


**Fig. S3. Concentration-response curve of dihydrokoumine in HEK293T cells using DMR assay.** Data are presented as mean ± SD (n = 3).


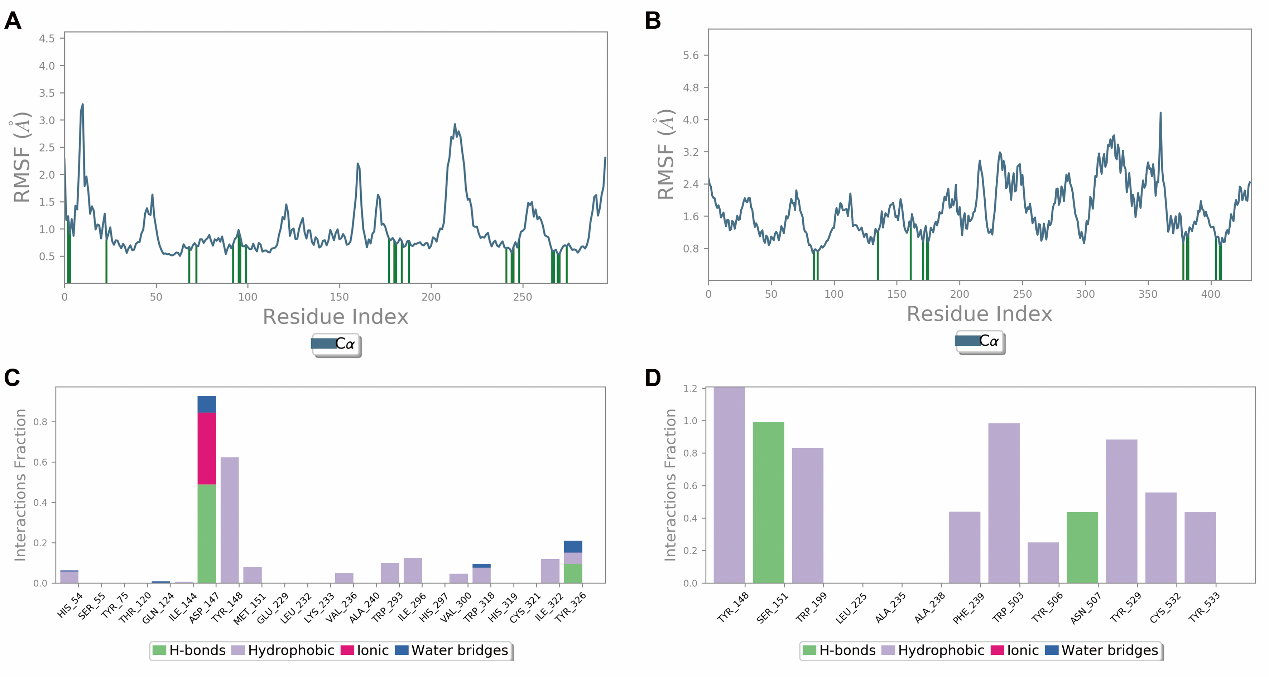


**Fig. S4.** **(A** and **B)** RMSF plots for the docked complex of MOR-DK (**A**) and M3R-DK (**B**). **(C** and **D)** Interactions fractions of protein-ligand complex of MOR-DK (**C** ) and M3R-DK (**D**).


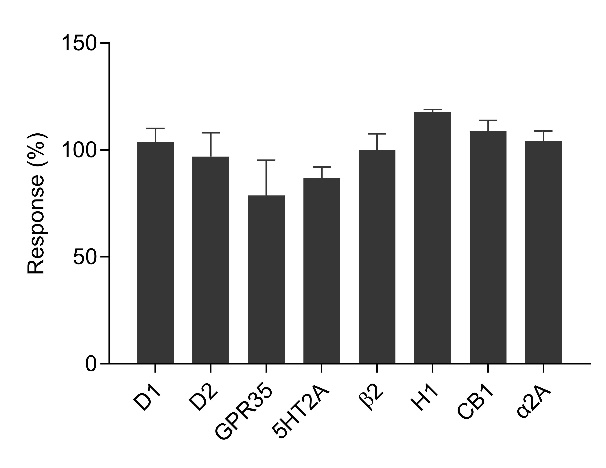


**Fig. S5. Receptor selectivity investigation of DK.** 100 μM DK was used to desensitize the DMR responses of D1 (dopamine), D2 (dopamine), GPR35 (zaprinast), 5HT2A (5-hydroxytryptamine), b2 (isoprenaline), H1 (histamine), CB1 (CP 55940) and α2A (L-Noradrenaline) probes. Data are presented as mean ± SD (n = 3).

**Fig. S6.** **The cumulative effects of naloxone on antinociception of DK in the hot plate assay** (morphine was 5 mg/kg, DK was 20 mg/kg, naloxone was 2 mg/kg). Data are presented as mean ± SEM (n = 6-8 mice per group). *P < 0.05, **P < 0.01, ***P < 0.001, ****P<0.0001 vs. the control group. #P < 0.05, ##P < 0.01, ###P < 0.001, ####P<0.0001 vs. the model group.


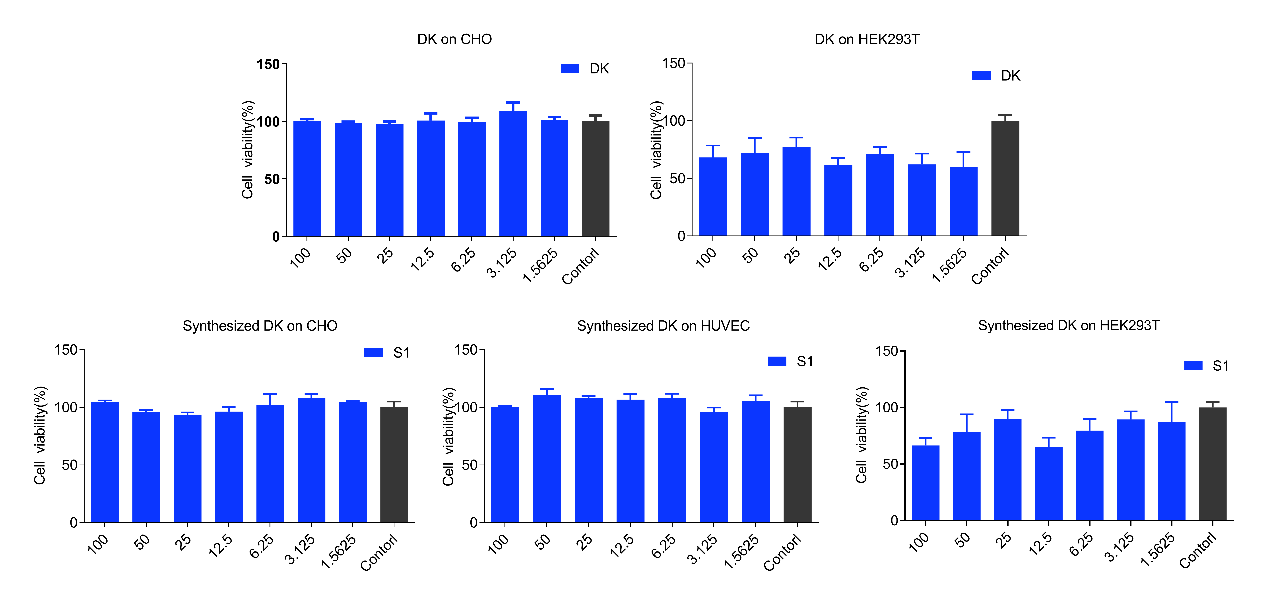


**Fig. S7.** **Cytotoxicity tests of purified DK and synthesized DK.** Cytotoxicity test of DK in CHO and HEK293T cells; cytotoxicity test of synthesized DK in CHO, HUVEC and HEK293T cells. Data are presented as mean ± SD (n = 3).

**
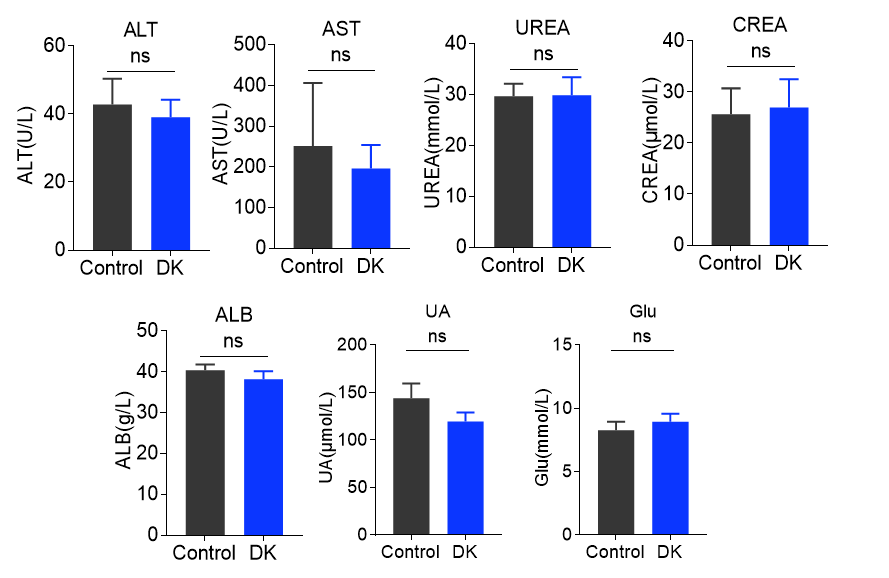
**

**Fig. S8. Effects of DK (200 mg/kg) on serum biochemical indicates in Mice.** Data are presented as mean ± SEM (n = 8).

**REFERENCES**

1 Zhang W, Zhang S-Y, Wang G-Y, Li N-P, Chen M-F, Gu J-H, et al. Five new koumine-type alkaloids from the roots of Gelsemium elegans*.* *Fitoterapia* 2017;**118**:112-7.
